# The Medico-Psychological Association and Their Nurses

**Published:** 1897-01-30

**Authors:** 


### EDITOR'S LETTER-BOX.

[Our Correspondents are reminded that prolixity is a great bar to publication, and that brevity of style and conciseness of statement greatly facilitate early insertion.]

### THE MEDICO-PSYCHOLOGICAL ASSOCIATION AND THEIR NURSES.

Dr. ERNEST WHITE, City of London Asylum, near Darenth, writes: As a member of the Educational Committee, and an examiner of the Medico-Psychological Association, I must regret the unsatisfactory and misleading nature of your editorial remarks appended to my letter of the 4th inst. You jump from the first edition of the Handbook, 1885, and the report of the committee of 1890, to the syllabus of the third edition of the Handbook of 1896, passing unnoticed the second edition, and the time interval, during which much progress was made. I must maintain that without the clinical instruction in the hospital and infirmary wards, where the practical examination is ultimately held, it is impossible to pass for the nursing certificate of the Association. Therefore the statement in your article that cramming by a book is sufficient is incorrect. The syllabus is ample for the requirements of the mental nurse. The public is at last realising that the hospital nurse of to-day is over-educated, and a possible source of danger. We are anxious that our mental nurses shall be efficient without intruding beyond their province.

"M. D." writes: Your remarks on the above subject in your issue of January 2nd are so full of common sense, and so much in agreement with practical experience, that I hope you will allow me to add one or two observations in support of your argument, and by way of a mild reminder to misguided beings that all is not as some would have us believe. May I venture to suggest that in the minds of many, if not the majority, there is a misconception as regards the opportunities afforded asylum nurses to become trained, experienced sick nurses. It would seem as if many overlooked the fact that in every asylum there is a huge hospital or infirmary block, and through which every nurse should pass. This means that the asylum nurse is primarily trained and taught to be a sick or hospital nurse, and, secondly, to be a mental nurse. This latter she cannot be under three years' training, so that while every asylum nurse can become an efficient hospital nurse as well as a mental nurse, the general hospital probationer or nurse has no opportunity of becoming a mental nurse, and it is well known how incompetent 99 per cent. of hospital nurses are when face to face with a mental case. I most heartily endorse and re-echo your sentiments condemnatory of the absurd and preposterous idea that responsible nursing posts in asylums should only be given to those who hold the certificate of the Medico-Psychological Association. No, no! much has been done, but much more remains to be done, and it is quite possible that a healthy difference of opinion, flavoured with a sprinkling of rivalry, will bear sound and ripe fruit in the proper season. As you rightly and truly state, hospitals train and certificate their own nurses, and no doubt each vies with the other to produce the best material, and so should it be with asylums. I must demur to the suggestion that the ordinary asylum nurse is inferior to the ordinary hospital nurse. In many asylums the training is equal to that given in hospitals, though I admit there is a difference of opinion regarding the value of the certificate, still I know of no reason why they should not rub shoulders as sister nurses on the register of the R.B.N.A., provided the executive is satisfied with the course of training and final examination. It is to be regretted that the Medico Psychological Association should be talked of as a social and administrative body, nothwithstanding that its list roll contains the names of the most eminent scientific men in the world. The letter of Dr. Ernest White in your last issue is no answer to your critical remarks, and obviously he answers himself.

Dr. G. Robertson, of Perth District Asylum, Murthly, writes: I take the liberty of encroaching on your space with my views of the difficulties created by the different status and training of hospital and mental nurses, to which you have given so much prominence, and the means I am adopting to overcome these. I do not intend to follow the side issue as to the other duties of the Medico-Psychological Association, but shall restrict myself to its aims for the welfare of our nurses and for the better nursing of our patients.

In the first place, your objection to the title mental nurse, as being misleading to many careless and uninformed people, is perfectly true; it might possibly have been better had a more distinctive title been adopted, though it is otherwise an excellent one, being perfectly accurate and very descriptive. However, the practical point to be fixed is this, as Dr. Wood has said: the title, whether one objects to it or not, exists, and, being a most suitable, short, and apt title, there is no power on the face of the earth that can taboo it and abolish its use. To attempt to do so would be a misguided policy, foredoomed to failure, and, as experience has taught us that when professions subdivide into special branches, the employment of titles becomes of necessity in course of time more exact and distinctive, so we must educate the careless public into a more accurate use of these titles. I have no fear that in a few years, by insisting on the use of the full title, hospital nurse and mental nurse, that the educated public will fully appreciate the difference of occupation, and that no professional harm will happen to the members of the R.B.N.A. Indeed, till then I apprehend a possibly different result, that hospital nurses may by mistake be called to attend mental cases, so that the title cuts both ways.

As for the training that our mental nurses undergo for the certificate of the Medico-Psychological Association, all no doubt pass through and receive practical instruction in the hospital ward of the asylum, but you wish to know if this can compare with that received by a hospital nurse either in quality or in point of time. Here and there, possibly, it may in the larger asylums, if the superintendent has interested himself in this question, but, on the whole, there can be no comparison. I would, however, ask the question, Why should there be anything like an equality of training in hospital nursing if there be a sufficiency? Our mental nurses do not profess to be hospital nurses, or to take their place, because they happen to be acquainted with the principles and practice of hospital nursing, in so far as these appertain to the treatment of mental disease. Our nurses profess to be mental nurses, and if they have obtained the certificate, those competent to judge consider that they are efficiently trained for the special work they profess. As skilled members of a special department of the great sisterhood of nursing, of which the nursing mother is the crown, these trained mental nurses desire to be registered in the Mental Branch of the R.B.N.A., and as they supply the most perfect mental nursing available, the R.B.N.A. would be neglecting its obvious duty to the public, who desire mental nursing, and would be setting back the hands of time, if it refused to recognise them. Mental nursing is as important a department of nursing as hospital nursing is, and makes greater demands upon qualities of the heart and of the head than the latter. When it is considered that a good mental nurse must be healthy of body and healthy of mind, intelligent and active, able to control herself with firmness and others with kindness; that she must be tactful, courteous, and conscientious; and when it is considered that her duties are confining and often irksome and and trying, it must be admitted that any woman of whom it can be truthfully said that she is a good mental nurse has, thereby, extensive testimony borne to her general excellence as a member of society. She has not received her just appreciation from the public, while her hospital sister is idolised by society. If, sir, Her Majesty's advisers could see their way in this year of jubilee to afford some recognition of mental nurses by her gracious patronage or otherwise, it would only be a just reward for long continued self-denial and devoted good work in the cold shade of neglect, and under circumstances the reverse of stimulating.

As to the special steps I have taken to meet some of the difficulties due to the different status and training of mental and hospital nurses, with the desire of bringing the nursing more into harmony, I have introduced trained hospital nurses among our pauper patients as staff nurses in charge of the wards. I hope by their precept and example to develop as much as much as possible and necessary hospital habits and nursing ideals, to raise the status of all the nurses, and the tone of the wards. This in course of time is bound to effect an improvement in the position of mental nurses and in the quality of hospital nursing in asylums, and perhaps overcome some of the defects in our training you have pointed out. In conclusion, while agreeing with my friend, Miss East, that hospital nurses are quite incompetent to take charge of insane cases, and no blame to them, for they receive absolutely no training in this important subject, I take exception to Dr. Urquhart's remarks. It is, perhaps, true that certain superintendents and asylum matrons have been unfavourably impressed by the assumption of superior knowledge by a certain type of hospital nurse, and even of a familiarity with insanity on the strength of a chance acquaintance with delirium, which really requires daily and long contact with the various types of mental disorder. The hospital nurse may be, and is, quite ignorant of insanity, but, being intelligent, educated, and trained to accurate observation, she makes first-class material for a mental nurse, and any "hysterical theory" that she may entertain can be knocked out of her head in five minutes. Why more hospital nurses do not go in for a training in lunacy as well I do not understand, as then many valuable posts would be open for them; and such additional training in asylum management and insanity as our hospital nurses are receiving here renders them specially eligible for the matronships of asylums.

